# A novel *GRN* mutation in an Italian patient with non-fluent variant of primary progressive aphasia at onset: a longitudinal case report

**DOI:** 10.3389/fnins.2023.1204504

**Published:** 2023-06-13

**Authors:** Veronica Castelnovo, Elisa Canu, Teuta Domi, Laura Pozzi, Francesca Vignaroli, Edoardo Gioele Spinelli, Alma Ghirelli, Giacomo Tondo, Cristoforo Comi, Nilo Riva, Angelo Quattrini, Paola Carrera, Massimo Filippi, Federica Agosta

**Affiliations:** ^1^Neuroimaging Research Unit, Division of Neuroscience, IRCCS San Raffaele Scientific Institute, Milan, Italy; ^2^Experimental Neuropathology Unit, Division of Neuroscience, IRCCS San Raffaele Scientific Institute, Milan, Italy; ^3^Movement Disorders Center, Neurology Unit, Department of Translational Medicine, University of Piemonte Orientale, Novara, Italy; ^4^Neurology Unit, IRCCS San Raffaele Scientific Institute, Milan, Italy; ^5^Vita-Salute San Raffaele University, Milan, Italy; ^6^Neurology Unit, S. Andrea Hospital, Department of Translational Medicine, University of Piemonte Orientale, Vercelli, Italy; ^7^Neurorehabilitation Unit, IRCCS San Raffaele Scientific Institute, Milan, Italy; ^8^Unit of Genomics for Human Disease Diagnosis, IRCCS San Raffaele Scientific Institute, Milan, Italy; ^9^Neurophysiology Service, IRCCS San Raffaele Scientific Institute, Milan, Italy

**Keywords:** progranulin, primary progressive aphasia, longitudinal, frontotemporal dementia, case report

## Abstract

**Objectives:**

We report the clinical presentation and evolution of a case with a novel Progranulin gene (*GRN*) mutation and non-fluent language disturbances at onset.

**Materials and methods:**

A 60 year-old, white patient was followed due to a history of language disturbances. Eighteen months after onset, the patient underwent FDG positron emission tomography (PET), and at month 24 was hospitalized to perform neuropsychological evaluation, brain 3 T MRI, lumbar puncture for cerebrospinal fluid (CSF) analysis, and genotyping. At month 31, the patient repeated the neuropsychological evaluation and brain MRI.

**Results:**

At onset the patient complained prominent language production difficulties, such as effortful speech and anomia. At month 18, FDG-PET showed left fronto-temporal and striatal hypometabolism. At month 24, the neuropsychological evaluation reported prevalent speech and comprehension deficits. Brain MRI reported left fronto-opercular and striatal atrophy, and left frontal periventricular white matter hyperintensities (WMHs). Increased CSF total tau level was observed. Genotyping revealed a new *GRN* c.1018delC (p.H340TfsX21) mutation. The patient received a diagnosis of non-fluent variant of primary progressive aphasia (nfvPPA). At month 31, language deficits worsened, together with attention and executive functions. The patient presented also with behavioral disturbances, and a progressive atrophy in the left frontal-opercular and temporo-mesial region.

**Discussion and conclusion:**

The new *GRN* p.H340TfsX21 mutation resulted in a case of nfvPPA characterized by fronto-temporal and striatal alterations, typical frontal asymmetric WMHs, and a fast progression toward a widespread cognitive and behavioral impairment, which reflects a frontotemporal lobar degeneration. Our findings extend the current knowledge of the phenotypic heterogeneity among *GRN* mutation carriers.

## Introduction

Progranulin gene (*GRN*) mutations are major causes of frontotemporal lobar degeneration (FTLD), occurring approximately in the 5–10% of FTLD cases (van Swieten and Heutink, 2008). This percentage increases to 22% in familial cases (van Swieten and Heutink, 2008). *GRN* mutations cause haploinsufficiency of progranulin, a protein involved in multiple neuroprotective functions, such as the promotion of neuron survival, neurite growth, and anti-inflammatory processes (van Swieten and Heutink, 2008).

To date, more than 114 *GRN* pathogenic variants have been identified (van Swieten and Heutink, 2008). The majority of mutation carriers develop a behavioral variant FTD (bvFTD) phenotype and another significant proportion of patients present with primary progressive aphasia (PPA), usually with the non-fluent variant (nfvPPA; van Swieten and Heutink, 2008). Patients present with apathy and social withdrawal as prominent behavioral changes (van Swieten and Heutink, 2008), while the most common cognitive features are language disturbances, such as effortful speech, word finding difficulties and phonemic paraphasias, and/or attention and executive dysfunctions. These cognitive alterations are followed by episodic memory and visuospatial deficits, limb apraxia, and dyscalculia (van Swieten and Heutink, 2008). Motor features, such as asymmetric parkinsonism and upper limb dystonia, have also been reported (van Swieten and Heutink, 2008). Compared to sporadic cases, *GRN* patients show severe and asymmetric brain atrophy with an early involvement of parietal lobes (van Swieten and Heutink, 2008).

## Case description

Here we report the clinical presentation and evolution over 3 years of a case with a novel *GRN* mutation and non-fluent language disturbances at onset. The proband is a 60 years-old white right-handed patient with 8 years of education, who at onset complained about prevalent language production difficulties, such as effortful speech and anomia. At that time, the patient was living alone and was still employed (although the work tasks of the patient were modified due to the cognitive symptoms). The proband had a positive family history for cerebrovascular diseases (mother).

**Figure 1:**
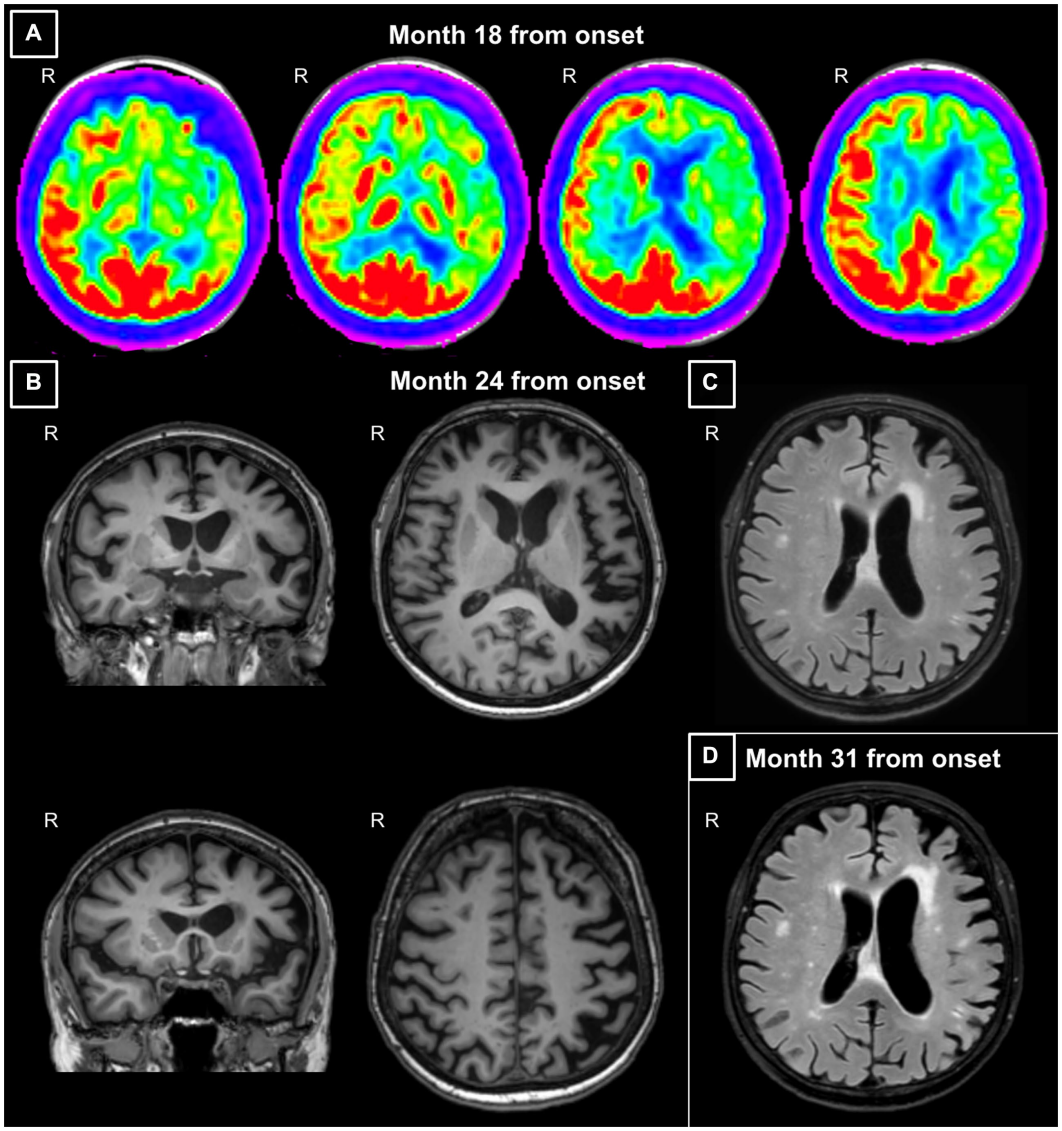
**(A)** Axial views of the fluorodeoxyglucose (FDG)-positron emission tomography (PET) acquired at month 18 from symptom onset. Images show hypometabolism in the left fronto-insular, fronto-temporal and temporo-lateral regions, and in the basal-ganglia with a left predominance. **(B)** Coronal and axial views of the 3DT1-weighted MR images acquired at month 24 from symptom onset. Images show left sylvian-perisylvian, fronto-opercular, parietal and striatal atrophy. **(C)** Axial view of the FLAIR image acquired at month 24 from symptom onset. The image shows prevalent left frontal periventricular white matter hyperintensities. **(D)** Axial view of the FLAIR image acquired at month 31 from symptom onset. The image shows a progressive ventricular enlargement mainly at the left side and increased burden of left frontal white matter hyperintensities. Images are displayed according to standard radiological convention. R, right.

After 1 year from the symptomatology onset, the patient underwent a speech/language evaluation which revealed the presence of anomia and phonemic paraphasias in speech production, mild difficulties in understanding syntactically complex sentences, and mild disturbances in computing calculations. This evaluation was followed by a 6-month cycle of speech/language training that the patient performed in a collaborative manner. A second speech/language assessment performed soon after the training showed the progression of language impairment and the appearance of agrammatism. After 18 months from symptom onset, the patient underwent a first neurological evaluation and was prescribed with an 18F-fluorodeoxyglucose positron emission tomography (FDG-PET). The neurological physical evaluation was normal and reported no focal neurologic signs, while the patient was spatially and temporally oriented and able to understand only syntactically simple commands. FDG-PET showed hypometabolism in the left fronto-insular, fronto-temporal, and temporo-lateral regions, and in the basal-ganglia with a left predominance (Figure 1A).

At 2 years from symptom onset, for further investigation, the patient was admitted to the local Neurology Unit to undergo computed tomography (CT), electroencephalogram (EEG), and cerebrospinal fluid (CSF) analysis. Brain CT revealed hypodensity of the periventricular white matter mainly at the level of the left frontal horn of the lateral ventricle, EEG did not show any abnormality, and CSF analysis showed slightly increased total tau level (489 pg./mL) with normal phosphorylated tau (34 pg./mL) and amyloid β42 levels (1,114 pg./mL). At the hospital discharge, the patient was addressed to a tertiary referral center for a comprehensive clinical and neuropsychological revaluation, 3 T brain MRI scan, and genotyping of the most common FTLD mutations (see Supplementary material for methodological details).

**Figure 2:**
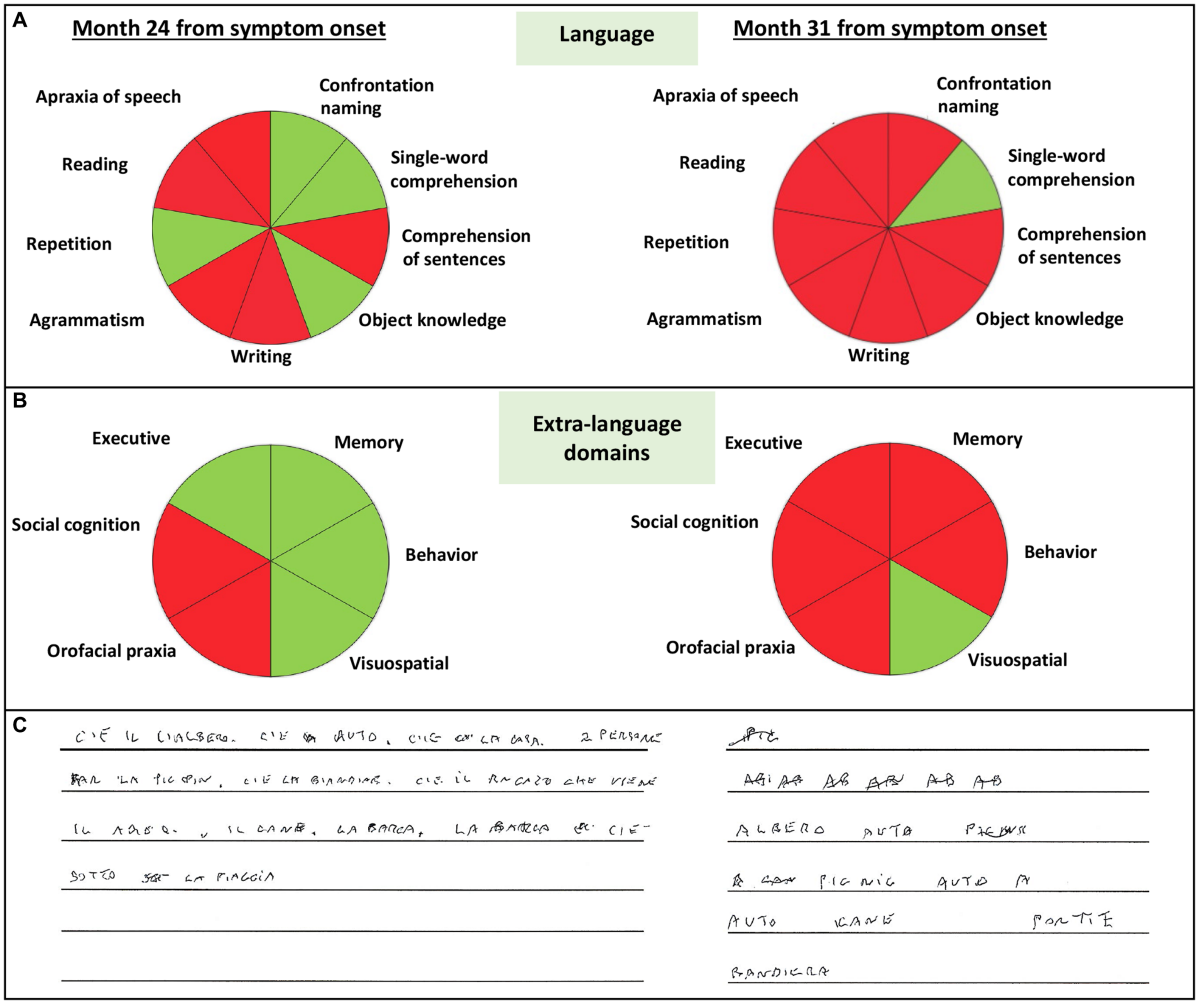
Schematic representation of impaired language **(A)**, extra-language domains **(B)**, and conducted writing **(C)** of the patient at month 24 and 31 from symptom onset. Green color indicates normal abilities, red color indicates impaired abilities.

At the tertiary referral center admission, the neurological evaluation confirmed the absence of focal motor and sensory deficits. The neuropsychological assessment confirmed a cognitive pattern still characterized by a predominant language impairment, with prevalent speech deficits, such as apraxia of speech and agrammatism, and altered sentence comprehension. At this time, the patient also showed mild non-linguistic deficits, such as orofacial apraxia, and difficulties in the attribution of others’ intentions and emotions (Table 1; Figure 2A). The insight was preserved, and no behavioral disturbances were refereed by the partner of the patient, except for moderate levels of anxiety. Brain 3 T MRI reported left sylvian-perisylvian, fronto-opercular, parietal, and striatal atrophy and prevalent left frontal periventricular white matter hyperintensities (WMHs; Figures 1B,C).

**Table 1:** Comprehensive neuropsychological assessment of the patient.

	Month 24 from symptoms’ onset			Month 31 from symptoms’ onset			Cut-off
	Raw	Adjusted	Presence of deficit	Raw	Adjusted	Presence of deficit	
Global cognition							
MMSE	21/30	19	*	−	−		<23.8
FAB	7/18	7.30	*	−	−		<13.5
Language							
*Confrontation naming*							
Confrontation naming, CaGi	45	44.76		17	16.76	*	≤41.48
*Single-word comprehension*							
Word-picture matching test, CaGi	48	48.04		48	48		≤47.08
*Syntactic comprehension*							
Token test	22	21.5	*	18	17.50	*	<26.50
*Object knowledge*							
Pyramids and palm tree test (visual)	50	49.62		35	35.03	*	≤40.15
*Repetition*							
Total repetition, AAT	146	−		65	−	*	≤142
*Reading*							
Reading, AAT	25	−	*	21	−	*	28
*Writing*							
Writing, AAT	21	−	*	18	−	*	27
*Apraxia of speech*	Present	−	*	Present	−	*	−
Memory							
Digit span, forward	3	3	*	NA	−		3.75
Spatial span, forward	4	4	+	2	1.75	*	3.75
Benson’s figure, recall	11	−		0	−	*	−
Benson’s figure, recognition	1	−		1	−		−
Executive functions							
Attentive matrices	39	38.25		14	10.75	*	<31
Digit span, backward	3	3.19	+	NA	−		2.65
MCST, categories	3	−	+	NA	−		3
Social cognition							
SET, global score	6	6.25	*	8	8.25	*	8.30
SET, intention attribution	2	2.17	*	1	1.17	*	<2.35
SET, emotion attribution	2	2.16	*	3	3.16	+	<2.21
Visuospatial abilities							
Benson’s figure, copy	16	−		16	−		−
Copy of simple figures	9	9.4		8	8.4		7.18
Praxia							
Orofacial apraxia, ideomotor	15	15	*	11	11	*	17
Ideomotor apraxia, right/left	20/20	−		20/18	−	+	17
Mood, autonomy, and disease severity							
Neuropsychiatric inventory	1	−		6	−		−
Frontal behavioral inventory, total	5	−		15	−		−
Frontal behavioral inventory, A	5			13			
Frontal behavioral inventory, B	0			2			
CDR	0	−		1	−		−
CDR, Sum of boxes	0	−		4	−		−
CDR, FTD	2	−		7.5	−		−
BADL	6/6	−		6/6	−		−
IADL	8/8	−		8/8	−		−

Cognitive scores are expressed as raw and adjusted scores (for age, education and sex when possible) according to the normative data (when available). ^*^pathological and ^+^borderline performances according to the normative data (see Supplementary material for details). AAT, aachener aphasie test; BADL/IADL, basic and instrumental activities of daily living; CDR, clinical dementia rating; FAB, frontal assessment battery; FTD, frontotemporal dementia; MCST, modified card sorting test; MMSE, mini mental state examination; NA, not applicable; and SET, story-based empathy task.

**Figure 3:**
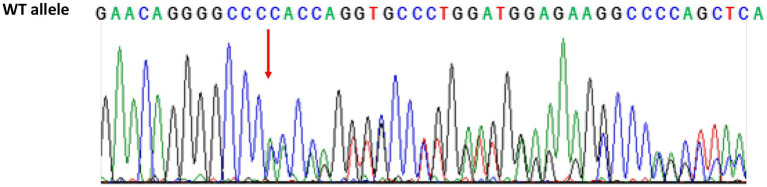
Sequencing chromatogram of *GRN* from the proband showing the heterozygous variant c.1018delC. The red arrow shows the C deletion in 1018 position in exon 10 introducing premature stop codon p.H340TfsX21 (*Homo sapiens*, Reference sequence ENSG00000030582.18, LRG_661). WT, wild type.

The genotyping revealed a new heterozygous *GRN* c.1018delC mutation (p.H340TfsX21), which determined the shift of amino acid 340 and, consequently, the introduction of a premature stop codon after 21 amino acids in the encoded protein of the *GRN* gene (Figure 3). According to the criteria of the American College of Medical Genetics (ACMG; Richards et al., 2015), this mutation is classified as likely pathogenetic. This variant is not present yet in the major genomic databases (Ensembl, GnomAd, and Exome Variant Server). The patient received a clinical, imaging-supported, and definite diagnosis of non-fluent variant of PPA (nfvPPA; Gorno-Tempini et al., 2011).

After 31 months from symptom onset, the patient underwent another neuropsychological assessment and a second 3 T MRI scan. Language deficits worsened, also including disturbances in single-word comprehension, object knowledge, and sentence repetition (see Figure 2C which reports the rapid progression of language impairment for the conducted writing). At this time, all extra-language domains except for visuospatial abilities were impaired (Table 1; Figure 2B), with a severe involvement of attention and executive functions. Furthermore, the partner of the patient referred moderate levels of apathy, aspontaneity, hyperorality, and preference for sweet foods. Compared to the first one, the second 3 T MRI examination revealed a progressive ventricular enlargement mainly at the left side with more atrophy of the left frontal-opercular and temporo-mesial regions, and increased burden of left frontal WMHs (Figure 1D).

## Discussion

Here, we reported the case of a 60 years old patient with a new *GRN* mutation characterized by a predominant language impairment for the first 2 years of the disease. The pattern of language deficits reflected a non-fluent aphasia with prevalent agrammatism in speech first and then in writing, apraxia of speech, and difficulties in sentence comprehension (Gorno-Tempini et al., 2011).

A PPA phenotype is not surprising in a case carrying a *GRN* mutation (van Swieten and Heutink, 2008). Specifically, sociodemographic and clinical profile of our case well reflects that of several *GRN* cases reported in the field. In a comprehensive description of a large case series, 18 *GRN* mutations (including seven novel ones) were identified in 32 *GRN* carriers (Le Ber et al., 2008). Among them, 5 (16%) presented with a clinical profile of PPA, with a mean age at onset of 59.8 years, with reduced fluency and/or language comprehension, and no behavioral disturbances during the first 2 years of the disease. In a previous report, we described a 63-year old patient carrying a *GRN* C157KfsX97 mutation, who, similar to the proband described in this report, presented severe apraxia of speech, mild agrammatism, occasional anomia, difficulty in understanding complex syntax, and moderate deficits in executive functions, without developing frank extrapyramidal symptoms during a 2-year follow-up (Caso et al., 2014). In another study, a 74-year-old patient carrying *GRN* p.P373RX37 mutation showed difficulties in naming, repetition and writing, and no alterations of behavior, executive functions, or memory within 1 year after diagnosis (Hosaka et al., 2017). Moreover, from a cohort of 45 *GRN*-mutated patients with PPA, authors identified nine nfvPPA patients with agrammatism and apraxia of speech as prominent features and a severe and rapid disease progression (Saracino et al., 2021). Finally, a previous investigation concerning FTD phenotypic studies on different pathogenic mutations has reported the same aminoacidic frameshift at protein level in GRN. This mutation was caused by a different nucleotide deletion on DNA sequence (c.1014del: p.His340Thrfs*21). The two patients included in the study belonged to two different families and had a diagnosis of bvFTD (Moore et al., 2020).

Interestingly, our patient presented early also with language features that are not typical of a non-fluent profile of PPA, such as the loss of word retrieval in spontaneous speech (anomia) and difficulties in reading and in writing under dictation. In sporadic cases, these features are observed in logopenic variants (Gorno-Tempini et al., 2011), but they are frequently reported in PPA cases with a *GRN* mutation (Rohrer et al., 2010). These language symptoms, together with other non-language disturbances (e.g., dyscalculia that our patient presented since the disease onset), are usually associated with brain damage involving the left parietal regions which is typical of *GRN* mutation (see details below; Rohrer et al., 2010).

Concerning non-language features, at the time of the diagnosis of nfvPPA, our patient had preserved insight and autonomy and did not show any behavioral disturbances. However, our proband was already presenting with difficulties in attributing others’ emotions and intentions. Although theory of mind abilities are understudied in PPA, they have been already reported in semantic and non-fluent cases at the disease onset or soon after 1 year of the disease course, and are beginning to be considered an early FTLD marker (Magno et al., 2022).

With the disease progression, our patient showed a worsening in attentive and executive functions and the appearance of behavioral changes. All these changes are expected to occur along with the progression of nfvPPA both in sporadic cases (Ulugut et al., 2022) and *GRN* carriers (Coppola et al., 2020). The rapid disease progression observed in our patient is typical of *GRN* cases (van Swieten and Heutink, 2008). Interestingly, we can speculate that the worsening of clinical symptoms in the first two years from disease onset was partially delayed by early speech/language training. In fact, in spite of the progressive nature of the disease, language stimulation programs applied in the early stages of the disease have proved to be effective in PPA (Pagnoni et al., 2021). However, as likely occurred in our patient, the long-term maintenance effects usually last till the end of the intervention (Pagnoni et al., 2021).

Brain structural MRI and FDG-PET images showed an asymmetric pattern of atrophy, with prevalent left side involvement, in frontal, temporal, parietal, and striatal regions. The asymmetric pattern of damage has been largely described in *GRN* mutations with PPA cases showing a prevalent left side involvement (Le Ber et al., 2008). Basal ganglia alterations were also expected given their presence in both symptomatic (Bocchetta et al., 2021) and pre-symptomatic cases carrying *GRN* mutation (Cash et al., 2018). During disease course, *GRN* carriers may present with parkinsonism (van Swieten and Heutink, 2008), a clinical sign typically associated with basal ganglia alterations. Although our proband did not present with motor symptoms, we should consider that, beyond motor control, basal ganglia play a fundamental role in speech articulation, executive functions and affective processing (Bocchetta et al., 2021), a cluster of cognitive domains which were found substantially impaired in our case. Finally, left frontal periventricular WMHs were also observed in our patient both using CT and MRI. The presence of asymmetric WMHs located in periventricular regions has been frequently reported in *GRN* mutation carriers and is considered a typical feature of *GRN* mutations in the absence of definite vascular risk factors (Le Ber et al., 2008; Rohrer et al., 2010; Caso et al., 2014; Coppola et al., 2020). In fact, progranulin lack has been associated with blood–brain barrier dysfunction and higher permeability, which have been shown to be associated with periventricular lesions (van Swieten and Heutink, 2008). However, WMHs could also be due to axonal degeneration (Woollacott et al., 2018). In particular, the severity of WMHs has been found to correlate with the location and extent of cortical atrophy (Woollacott et al., 2018), and brain regions with the most severe WMH have been shown to display severe white matter pathology on histological analysis (Woollacott et al., 2018).

In conclusion, the new *GRN* p.H340TfsX21 mutation resulted in a case of nfvPPA characterized by speech production and comprehension deficits, fronto-insular, temporal and striatal hypometabolism and atrophy, typical frontal asymmetric WMHs, and a fast progression toward a widespread cognitive and behavioral impairment. Although the study has some limitations, such as the lack of a longer follow-up, a detailed family history, and a confirmed neuropathology, this is the first report of the occurrence of the *GRN* p.H340TfsX21 mutation in an Italian case with PPA. Our findings extend the current knowledge of the phenotypic heterogeneity among *GRN* mutation carriers.

## Data availability statement

The original contributions presented in the study are included in the article/Supplementary material, further inquiries can be directed to the corresponding author.

## Ethics statement

The studies involving human participants were reviewed and approved by ethical standards committee on human experimentation of IRCCS San Raffaele Scientific Institute. The patients/participants provided their written informed consent to participate in this study. Written informed consent was obtained from the individual(s) for the publication of any potentially identifiable images or data included in this article.

## Author contributions

VC, EC, MF, and FA conceived and designed the study. TD, LP, FV, ES, AG, GT, CC, NR, AQ, and PC acquired data. VC, EC, TD, NR, and LP analyzed data. All authors contributed to the article and approved the submitted version.

## Funding

This study has been supported by the European Research Council (StG2016_714388_NeuroTRACK) and the Foundation Research on Alzheimer Disease.

## Conflict of interest

EC has received research supports from the Italian Ministry of Health. MF received compensation for consulting services from Alexion, Almirall, Biogen, Merck, Novartis, Roche, and Sanofi; speaking activities from Bayer, Biogen, Celgene, Chiesi Italia SpA, Eli Lilly, Genzyme, Janssen, Merck-Serono, Neopharmed Gentili, Novartis, Novo Nordisk, Roche, Sanofi, Takeda, and TEVA; participation in Advisory Boards for Alexion, Biogen, Bristol-Myers Squibb, Merck, Novartis, Roche, Sanofi, Sanofi-Aventis, Sanofi-Genzyme, and Takeda; scientific direction of educational events for Biogen, Merck, Roche, Celgene, Bristol-Myers Squibb, Lilly, Novartis, and Sanofi-Genzyme; and received research support from Biogen Idec, Merck-Serono, Novartis, Roche, Italian Ministry of Health, and Fondazione Italiana Sclerosi Multipla. FA has received speaker honoraria from Biogen Idec, Roche, Zambon, and Italfarmaco; and receives or has received research supports from the Italian Ministry of Health, Italian Ministry of University and Research, AriSLA (Fondazione Italiana di Ricerca per la SLA), the European Research Council, and the Foundation Research on Alzheimer Disease.

The remaining authors declare that the research was conducted in the absence of any commercial or financial relationships that could be construed as a potential conflict of interest.

## Publisher’s note

All claims expressed in this article are solely those of the authors and do not necessarily represent those of their affiliated organizations, or those of the publisher, the editors and the reviewers. Any product that may be evaluated in this article, or claim that may be made by its manufacturer, is not guaranteed or endorsed by the publisher.
