# Supplementary material for: A novel GRN mutation in an Italian patient with non-fluent variant of primary progressive aphasia at onset: a longitudinal case report
Source: Front Neurosci. 2023 Jun 13;17:1204504. doi: 10.3389/fnins.2023.1204504 (PMC10296183; doi:10.3389/fnins.2023.1204504)
Supplement: Supplementary file 1 [file Data_Sheet_1.docx]

**Neuropsychological assessment**

At the tertiary referral center, a comprehensive neuropsychological assessment was performed at month 24 and month 31 from symptom onset by an experienced neuropsychologist and evaluated: global cognitive functioning with the Mini-Mental State Examination (1); frontal functioning with the Frontal Assessment Battery (2); verbal memory with the digit span forward (3); non-verbal memory with the spatial span forward (3), and Benson’s Figure delayed recall (4); attention and working memory with the attentive matrices (5), the digit span backward (6), and the Modified Card Sorting Test (7); visuospatial abilities with the Benson’s figure copy (4) and the copy of simple figures (8); praxia with orofacial and ideomotor limb apraxia tests (5); social cognition with the Story-based Empathy Task (9); behaviour with the Neuropsychiatric inventory (10) and the Frontal Behavioural inventory (11); disease severity with the clinical dementia rating scales (12, 13), and autonomy with basic (14) and instrumental (15) activities of daily living.

The patient also underwent a comprehensive language testing which evaluated: syntactic comprehension with the Token test (16); confrontation naming and single word comprehension with the CaGi test (17); object knowledge with the Pyramids and Palm Trees Test (18); repetition, reading, and writing with the Aachener Aphasie Test (19). For investigating motor speech and speech fluency, we recorded, respectively, multiple repetitions of single words (not standardized) and speech samples while the patient described the image of the picnic picture subtest of the Western Aphasia Battery (20).

**MRI acquisition**

MRI acquisition was performed at the tertiary referral center. Using a 3.0 T scanner (Ingenia CX, Philips), the following brain MRI sequences were obtained at month 24 and month 31: 3D T1-weighted (TFE) (TR=7 ms; TE=3.2 ms; flip angle=9 [degrees]; 204 contiguous sagittal slices with voxel size=1 x 1 x 1 mm, matrix size=256 x 240, FOV=256x240 mm2); 3D FLAIR (TR=4800 ms; TE=267 ms; TI=1650 ms; ETL=167; NEX=2; 192 contiguous sagittal slices with voxel size=0.89 x 0.89 x 1 mm, matrix size=256 x 256, FOV=256x256 mm2); and 3D T2 (TR=2500 ms; TE=330 ms; ETL=117; NEX=1; 192 contiguous sagittal slices with voxel size=0.89 x 0.89 x 1 mm, matrix size=256 x 258, FOV=256x256 mm2).

**Progranulin sequencing**

At month 24 total genomic DNA was extracted from peripheral blood using NucleoSpin Blood L Extraction Kit (Macherey-Nagel) following the manufacturer instructions. *GRN* (Homo sapiens, ENSG00000030582.18, LRG_661) whole coding region and the intron-exon junctions were analyzed with a Sanger protocol (21).

**References**

1. Folstein MF, Folstein SE, McHugh PR. "Mini-mental state". A practical method for grading the cognitive state of patients for the clinician. J Psychiatr Res. 1975;12(3):189-98.

2. Appollonio I, Leone M, Isella V, Piamarta F, Consoli T, Villa ML, et al. The Frontal Assessment Battery (FAB): normative values in an Italian population sample. Neurol Sci. 2005;26(2):108-16.

3. Orsini A, Grossi D, Capitani E, Laiacona M, Papagno C, Vallar G. Verbal and spatial immediate memory span: normative data from 1355 adults and 1112 children. Ital J Neurol Sci. 1987;8(6):539-48.

4. Possin KL, Laluz VR, Alcantar OZ, Miller BL, Kramer JH. Distinct neuroanatomical substrates and cognitive mechanisms of figure copy performance in Alzheimer's disease and behavioral variant frontotemporal dementia. Neuropsychologia. 2011;49(1):43-8.

5. Spinnler H, Tognoni G. Standardizzazione e taratura italiana di test neuropsicologici. Ital J Neurol Sci. 1987;6, suppl 8:44-6.

6. Monaco M, Costa A, Caltagirone C, Carlesimo GA. Forward and backward span for verbal and visuo-spatial data: standardization and normative data from an Italian adult population. Neurol Sci. 2013;34(5):749-54.

7. Caffarra P, Vezzadini G, Dieci F, Zonato F, Venneri A. Modified Card Sorting Test: normative data. J Clin Exp Neuropsychol. 2004;26(2):246-50.

8. Carlesimo GA, Caltagirone C, Gainotti G. The Mental Deterioration Battery: normative data, diagnostic reliability and qualitative analyses of cognitive impairment. The Group for the Standardization of the Mental Deterioration Battery. Eur Neurol. 1996;36(6):378-84.

9. Dodich A, Cerami C, Canessa N, Crespi C, Iannaccone S, Marcone A, et al. A novel task assessing intention and emotion attribution: Italian standardization and normative data of the Story-based Empathy Task. Neurol Sci. 2015;36(10):1907-12.

10. Cummings JL, Mega M, Gray K, Rosenberg-Thompson S, Carusi DA, Gornbein J. The Neuropsychiatric Inventory: comprehensive assessment of psychopathology in dementia. Neurology. 1994;44(12):2308-14.

11. Alberici A, Geroldi C, Cotelli M, Adorni A, Calabria M, Rossi G, et al. The Frontal Behavioural Inventory (Italian version) differentiates frontotemporal lobar degeneration variants from Alzheimer's disease. Neurol Sci. 2007;28(2):80-6.

12. Borroni B, Agosti C, Premi E, Cerini C, Cosseddu M, Paghera B, et al. The FTLD-modified Clinical Dementia Rating scale is a reliable tool for defining disease severity in frontotemporal lobar degeneration: evidence from a brain SPECT study. Eur J Neurol. 2010;17(5):703-7.

13. Hughes CP, Berg L, Danziger WL, Coben LA, Martin RL. A new clinical scale for the staging of dementia. Br J Psychiatry. 1982;140:566-72.

14. Katz S, Ford AB, Moskowitz RW, Jackson BA, Jaffe MW. Studies of Illness in the Aged. The Index of Adl: A Standardized Measure of Biological and Psychosocial Function. JAMA. 1963;185:914-9.

15. Lawton MP, Brody EM. Assessment of older people: self-maintaining and instrumental activities of daily living. Gerontologist. 1969;9(3):179-86.

16. De Renzi E, Vignolo LA. The token test: A sensitive test to detect receptive disturbances in aphasics. Brain. 1962;85:665-78.

17. Catricala E, Della Rosa PA, Ginex V, Mussetti Z, Plebani V, Cappa SF. An Italian battery for the assessment of semantic memory disorders. Neurol Sci. 2013;34(6):985-93.

18. Gamboz N, Coluccia E, Iavarone A, Brandimonte MA. Normative data for the Pyramids and Palm Trees Test in the elderly Italian population. Neurol Sci. 2009;30(6):453-8.

19. Luzzatti C, Willmes K, De Bleser R, Bianchi A, Chiesa G, De Tanti A, et al. New normative data for the Italian version of the Aachen Aphasia Test (A.A.T.) Archivio di Psicologia Neurologia e Psichiatria. 1994;55(6):1086-131.

20. Kertesz A. Western Aphasia Battery. New York: Grune & Stratton; 1982.

21. Pozzi L, Valenza F, Mosca L, Dal Mas A, Domi T, Romano A, et al. TBK1 mutations in Italian patients with amyotrophic lateral sclerosis: genetic and functional characterisation. J Neurol Neurosurg Psychiatry. 2017;88(10):869-75.
